# RTS Noise and Dark Current White Defects Reduction Using Selective Averaging Based on a Multi-Aperture System

**DOI:** 10.3390/s140101528

**Published:** 2014-01-16

**Authors:** Bo Zhang, Keiichiro Kagawa, Taishi Takasawa, Min Woong Seo, Keita Yasutomi, Shoji Kawahito

**Affiliations:** Research Institute of Electronics, Shizuoka University, 3-5-1 Johoku Nakaku Hamamatsu, Shizuoka 432-8011, Japan; E-Mails: kagawa@idl.rie.shizuoka.ac.jp (K.K.); ttakasawa@idl.rie.shizuoka.ac.jp (T.T.); mwseo@idl.rie.shizuoka.ac.jp (M.W.S.); kyasu@idl.rie.shizuoka.ac.jp (K.Y.); kawahito@idl.rie.shizuoka.ac.jp (S.K.)

**Keywords:** noise reduction, multi-aperture, random telegraph signal (RTS) noise, dark current white defect

## Abstract

In extremely low-light conditions, random telegraph signal (RTS) noise and dark current white defects become visible. In this paper, a multi-aperture imaging system and selective averaging method which removes the RTS noise and the dark current white defects by minimizing the synthetic sensor noise at every pixel is proposed. In the multi-aperture imaging system, a very small synthetic F-number which is much smaller than 1.0 is achieved by increasing optical gain with multiple lenses. It is verified by simulation that the effective noise normalized by optical gain in the peak of noise histogram is reduced from 1.38e^-^ to 0.48e^-^ in a 3 × 3-aperture system using low-noise CMOS image sensors based on folding-integration and cyclic column ADCs. In the experiment, a prototype 3 × 3-aperture camera, where each aperture has 200 × 200 pixels and an imaging lens with a focal length of 3.0 mm and F-number of 3.0, is developed. Under a low-light condition, in which the maximum average signal is 11e^-^ per aperture, the RTS noise and dark current white defects are removed and the peak signal-to-noise ratio (PSNR) of the image is increased by 6.3 dB.

## 1. Introduction

Recently, CMOS image sensors (CISs) have become wildly used in scientific, industrial, and biomedical applications, as well as consumer cameras. Noise is one of the crucial elements which limit the performance of CISs. Especially in low-light applications, the sensor noise such as dark current white defects and amplifier noise becomes more visible. Since the active pixel sensor (APS) with pinned photodiode technology was applied in CISs in the mid 1990's, the reset and dark current in CISs have been improved significantly [1,2]. In the last two decades, many methods have been presented for low-noise CIS design. High-gain amplifiers [3–5] for reducing readout circuits noise and amplifiers with multiple sampling [6–12] are effective for reducing thermal noise of readout circuits. However, these high gain amplifiers are not useful for reducing the in-pixel random telegraph signal (RTS) noise and dark current white defects.

RTS noise is described as a fluctuation in the current of a MOSFET [13] and it is generated by capturing and emission of carriers in the MOSFET channel randomly at the traps of the silicon-silicon dioxide interface. The RTS noise in CISs is a major issue, especially in low-light applications [14]. As the transistor is scaled down, the impurity concentration in the channel becomes non-uniform, and large RTS noise will appear [15]. The RTS noise can be reduced by the in-pixel buried channel source follower [16], in which an n-type doping along the channel of n-MOS transistor is introduced to lead the channel away from the Si-SiO_2_ interface. Thus, the influence of traps near the interface is alleviated, so the 1/f and RTS noise can be reduced. However, the buried channel causes lower transconductance, which leads to larger thermal noise.

In this paper, the RTS noise and dark current reduction based on a multi-aperture imaging system with a selective averaging method is presented. The multi-aperture system is composed of multiple components for both lens and sensor. In the proposed method, the multiple lenses are regarded as a synthetic single lens to collect more photons. The multiple pixels of each aperture that correspond to a certain reproduced pixel are treated as a sub-pixel. Namely, every virtual pixel is composed of real sub-pixels. Such redundancy is utilized to reduce the sensor noise. In the noise reduction method, the apertures are selected to minimize a combination variance of pixel value for each pixel in the dark condition. During capturing images, the pixel values among the selected apertures are averaged to calculate the pixel value of the reproduced final image.

The rest of this paper is organized as follows: in Section 2 the fundamentals of the multi-aperture imaging system and the principle of selective averaging method are described. SNR in the multi-aperture system is discussed in Section 3. Section 4 shows the simulation results. The image processing procedure in the multi-aperture noise reduction and results are shown in Section 5. Section 6 gives the conclusions.

## 2. Noise Reduction by Selective Averaging in Multi-Aperture System

### 2.1. Multi-Aperture System

**Figure 1.**
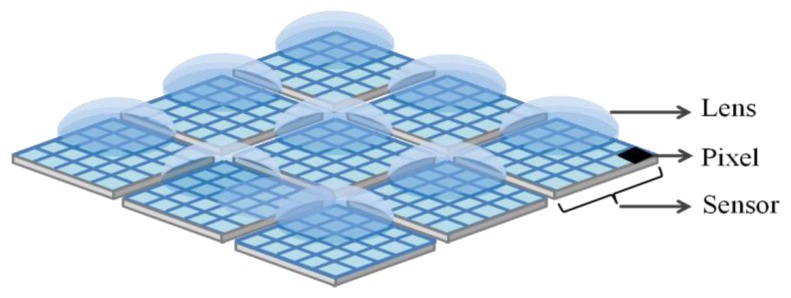
Architecture of multi-aperture imaging system.

The most different feature of the multi-aperture system compared to traditional single cameras is that in the multi-aperture system [17] multiple compact lenses and sensors in an N × N array are utilized. Note that each lens has a corresponding sensor like a traditional single-aperture camera. Here, it is assumed that the pixel sizes for the apertures are the same, and each aperture acquires a complete image. Figure 1 shows the architecture of multi-aperture system. As shown in [17], for example, the multi-aperture system is used to increase resolution, dynamic range, and frame rate. However, we apply it to reduce RTS noise and dark current white defects.

### 2.2. Merits of Multi-Aperture System

**Figure 2.**
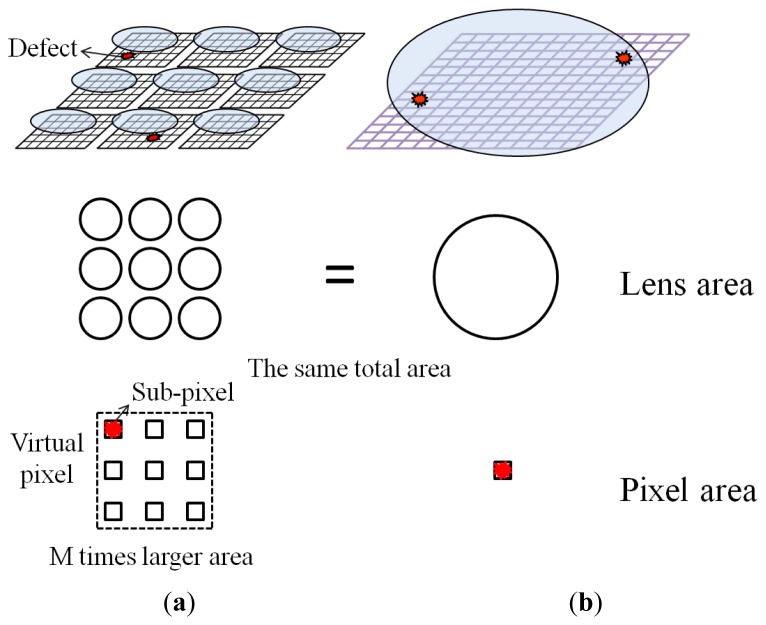
Comparison of (**a**) multi-aperture camera and (**b**) single aperture counterpart.

To increase the SNR of acquired images under low-light conditions for the given accumulation time determined by a frame rate, a smaller F-number lens is necessary. In the conventional single-aperture camera, the small F-number lens has a large pupil. To show the effectiveness of the multi-aperture camera, this is compared with a single aperture counterpart shown in Figure 2b. The single aperture counterpart is an ideal conventional camera whose F-number is the same as the synthetic F-number of multi-aperture system as shown in Figure 2a. In the proposed method, M pixels from every aperture are regarded as a sub-pixel, and they emulate a large pixel whose area is M times larger than that of the single aperture counterpart. Therefore, the number of pixels in the reproduced image out of the multi-aperture camera is as many as N × N although the number of physical pixels is M × N × N in the proposed method. To correct the lens aberration, more lens elements are required, so that the lens group will become very large and heavy. For example, when we consider Canon's commercial fixed-focal lenses with a focal length of 50 mm, an F/1.8 lens weighs only 130 g. However, the weight of a F/1.2 lens can be as much as 590 g [18]. In the multi-aperture system, the number of incident photons is multiplied by the number of lenses in the lens array. It can be considered that the synthetic F-number (F_s_) in the multi-aperture system becomes ${\text{F}}_{0}/\sqrt{\text{M}}$ where F_0_ is the F-number of elemental lens. When we think of realizing a synthetic F-number of 1.2 (F_s_ = 1.2) with multiple F/1.8 (F_0_ = 1.8) lenses in the multi-aperture system, M equals 2.25 (M = (F_0_/F_s_)^2^). Therefore, the weight of the virtual F/1.2 multi-aperture system becomes 292.5 g (= 130 g × 2.25). The F/1.2 single-aperture lens is about twice as heavy as the multi-aperture counterpart.

Another virtue of multi-aperture image system is that defect pixels are removed in the reproduced image without any interpolation. As shown in Figure 2, in general, there are multiple malfunctioning pixels on a CIS due to fabrication errors, which cause permanent dark, bright, or blinking dots in images, and degrade the image quality. In the multi-aperture imaging system, there are multiple pixels (N × N) for one identical objective point. If one aperture has a defective pixel, the pixels of the other apertures can be used to calculate the pixel value of the unified image.

In low-light applications, because the signal level is very low, the RTS and dark current white defects become clearly visible, which seriously degrades the quality of image. Such large noise can be removed by the multi-aperture system with a selective averaging method discussed below.

### 2.3. Selective Averaging Method

In the multi-aperture imaging system, *N* × *N* images for one picture are acquired simultaneously. However, the pixel values for an identical objective point are not exactly the same, because different random noise of pixel amplifier and ADC is added to each of them.

**Figure 3.**
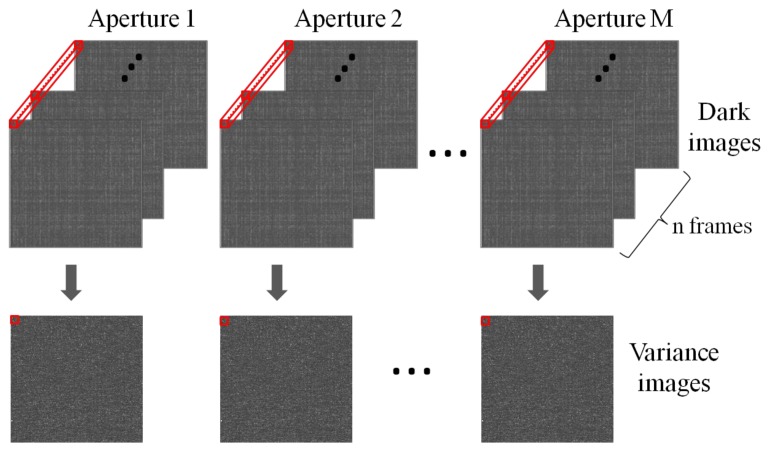
Variance calculations in multi-aperture camera.

Figure 3 shows the variance calculation in the multi-aperture imaging system, where *n*-frame images are captured in a dark condition. Obviously, there are *N* × *N* variances for one objective point and the values of variance in the apertures are different pixel to pixel. The variances are sorted from the minimum to the maximum for each pixel, and then a combination variance is calculated by using the following equation:

(1) $$S_{m}^{2}=\frac{1}{m^{2}}\begin{array}{ll} \sum_{i=1}^{m}\sigma_{i}^{2} & \left(1\leq m\leq N^{2}\right) \end{array}$$

here, *m* is the number of the selected apertures, $\sigma_{i}^{2}$ is a sorted variance, and $S_{m}^{2}$ is the combination variance.

The sorting process is to find the smallest combination variance $S_{m}^{2}$ among *m* apertures. Generally, as *m* increases, the combination variance becomes smaller according to the factor of 1/*m*^2^, when the variances are comparable. Sometimes pixels have very large noise due to RTS noise or shot noise of large dark current, so that the variance of these pixels will be very large. If the variance is relatively large, the combination variance for (*m* + 1) apertures can be larger than the combination variance for *m* apertures. Thus, pixels with large noise are automatically excluded.

**Figure 4.**
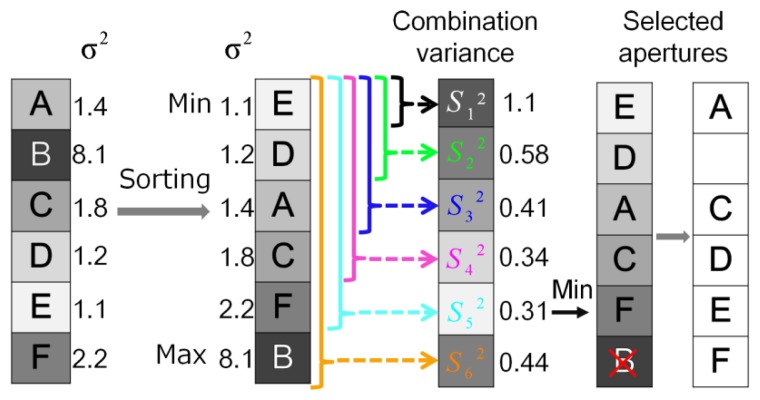
Procedure of aperture selection for one pixel.

Figure 4 shows an example of aperture selection at one pixel. The variance in aperture B is much larger than the others, and it causes that the combination variance $S_{6}^{2}$ is larger than $S_{5}^{2}$. The minimum combination variance is found out to be $S_{5}^{2}$, and the apertures used to calculate the minimum combination variance can also be determined as A, C, D, E, and F. Those apertures are the selected apertures for this pixel. In shooting images, the pixel values among the selected apertures are averaged, and the averaged value is defined as the pixel value of the unified virtual image. Because averaging is operated only to the selected apertures, this method is called selective averaging. When the RTS noise and the shot noise caused by dark current are very large, it causes a large variance. In this method, the apertures that have a large variance will not be selected, so these noise components can be removed.

For some of the dark or bright defect pixels, the pixel values can be continuously very small or large, respectively, but the variance is small. Such situation can happen when the pixel source followers do not work correctly. If the selective averaging method is applied directly, these pixel values of the reproduced image will be deteriorated. In this situation, thresholding is effective. If the minimum pixel value for *n*-frame images (Figure 3) is larger than the threshold or pixel value is insensitive to light, we assign a huge number to the variance of these defect pixels, so that those will not be selected.

## 3. SNR Analysis in Multi-Aperture System

In this session, SNRs of the conventional single-aperture and multi-aperture cameras with the selective averaging are compared based on a simple noise model. The SNR of conventional single aperture system, where sensor noise and photon shot noise are considered, is given by:

(2) $${\text{SNR}}_{SA}=20\log_{10}\frac{N_{e}}{\sqrt{\sigma_{\text{sensor}}^{2}+N_{e}}}$$

here *N_e_* is the number of signal electrons, $\sigma_{\text{sensor}}^{2}$ is a variance of the input referred sensor noise in electron.

In the multi-aperture system, because multiple apertures are used, its SNR with summing up *m* apertures for averaging is given by:

(3) $${\text{SNR}}_{MA}=20{\text{log}}_{10}\frac{m\cdot N_{e}}{\sqrt{m\cdot \sigma_{\text{sensor}}^{2}+m\cdot N_{e}}}=20{\text{log}}_{10}\frac{\sqrt{m}\cdot N_{e}}{\sqrt{\sigma_{\text{sensor}}^{2}+N_{e}}}$$

Actually, in the selective averaging method, the number of selected apertures, *m*, varies from 1 to M. *N_e_* has a relationship with illumination and some other parameters, which is given by:

(4) $$N_{e}=a\cdot \eta \cdot A\cdot \frac{RT}{4F_{n}^{2}}\cdot E_{o}\cdot \tau$$

where *a* is a proportional constant, *η* is the quantum efficiency, which is the ratio of the number of generated electrons to the number of incident photons, *A* is pixel area, *R* is object reflectivity, *T* is lens transmittance, *F_n_* is F-number of a single aperture, which is calculated by the ratio of focal length *f* to aperture diameter *D*, *E_o_* is illumination on the object surface, and *τ* is exposure time.

To simplify the equation, we define the product of the *a*, *η*, *R*, *T*, *E_o_* and *τ* as a constant *C*, and Equation (4) is expressed as:

(5) $$N_{e}=C\cdot \frac{A}{F_{n}^{2}}$$

Using Equations (2) and (4) this becomes:

(6) $${\text{SNR}}_{MA}=20{\text{log}}_{10}\frac{\sqrt{m}\cdot C\cdot A/F_{n}^{2}}{\sqrt{\sigma_{\text{sensor}}^{2}+C\cdot A/F_{n}^{2}}}$$

Equation (6) shows the relationship between SNR, pixel area, and F-number. To increase the SNR, the F-number should be reduced or the pixel area increased. If we reduce the F-number, the diameter of the aperture should increase. However, in fact, it is not easy to realize a compact and fast lens with a high spatial resolution. Another way to increase the SNR is to increase the pixel area. When the field of view is fixed, this option requires a larger image circle, which does not mean to require a high spatial resolution, but to make the lens bigger and heavier. Therefore, in the camera design, taking the balance of pixel area and F-number is crucial.

To compare the performance of the single-aperture and multi-aperture cameras, the multi-aperture camera should be compared with its single aperture counterpart shown in Figure 2b, which has the same F-number as that of the synthetic F-number of the multi-aperture camera. The SNR of the single aperture counterpart is written by:

(7) $${\text{SNR}}_{\text{SAPCP}}=20{\text{log}}_{10}\frac{M\cdot N_{e}}{\sqrt{\sigma_{\text{sensor}}^{2}+M\cdot N_{e}}}$$

The signal levels are the same for the multi-aperture system and the signal aperture counterpart when m = M. However, the noise factor in Equation (3) is m times as large as that in Equation (7), which is caused by the number of physical pixels related to a single reproduced pixel. The sensor noise (*σ*_sensor_) is summed up among m pixels in the multi-aperture system. On the other hand, in the single aperture counterpart, only one sensor is used, so the noise factor is unity when we assume that the sensor noise of single aperture counterpart and that of each aperture are the same.

**Figure 5.**
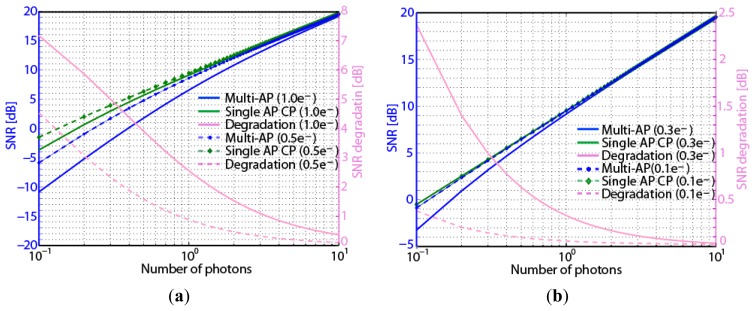
SNRs for different values of amplifier noise. (**a**) 1.0e^-^ and 0.5e^-^. (**b**) 0.3e^-^ and 0.1e^-^. (AP: aperture, CP: counterpart).

Figure 5a shows the calculation results of SNR of multi-aperture and single aperture counterpart when the number of apertures is 9 (*m* = 9), and the average number of photons varies from 10^−1^ to 10^1^ for the sensor noise of 1.0e^-^ and 0.5e^-^.

The SNR of the multi-aperture is obviously smaller when the number of photons is below 10^1^. The degradation is shown by the difference between the SNRs for the multi-aperture and the single aperture counterpart. Because the noise power of the multiple apertures is summed up, the SNR for the multi-aperture camera is smaller than that for the single lens counterpart. As the number of photons is increased, the photon shot noise also increases. Because the level of the sensor noise is independent of the incident light intensity and the photon shot noise becomes the dominant noise when incident light is strong enough, the effect of sensor noise can be negligible in SNR calculation. So the SNR of multi-aperture can be considered to be almost the same as that of the single aperture counterpart when the number of photons is larger than 10^1^.

Figure 5b shows the calculated SNRs for smaller sensor noise such as 0.3e^-^ and 0.1e^-^. When the sensor noise is less than 0.3e^-^, the SNRs of multi-aperture and single aperture counterpart can be considered to be almost the same even in the very low light conditions.

## 4. Simulation Results

As shown in Figure 1, the multiple image sensors are utilized in the multi-aperture camera, and all apertures work synchronously. However, in the following simulations and experiments, a 3 × 3 lens array is put on a single CIS to emulate a multi-aperture system. The CIS has 1280(*V*) × 1024(*H*) pixels, and is fabricated in 0.18 *μm* 1-poly 4-metal CIS process with the pinned photodiode [12]. The image from the CIS is separated into nine regions by the lens array. The pixel count in each region is 200 × 200. The data is processed by MATLAB.

In the simulation, the selective averaging method is compared with the single aperture counterpart, single region image (referred as “raw”), and some other processing methods in the multi-aperture system such as simple averaging, minimum selection, and median selection. In the simple averaging, no selection is done. All the apertures are used to take average. Minimum selection means the aperture with the minimum variance is selected for every pixel, and the pixel value of the selected aperture is copied to the final reproduced image. In median selection, the median pixel value among the multiple apertures is calculated for every pixel.

To compare six cases, the resultant noise is normalized by optical gain. The optical gain is introduced to compare the S/Ns of different optical configurations and processing methods. Here, optical gain is defined by the ratio of the number of incident photons, namely effective pupil area, based on that of “raw”. The optical gains for raw, minimum selection, and median selection are all 1 because the pixel value of only one aperture is used. The optical gain for the simple averaging is 9, which is the same as the number of apertures (M). For the single aperture counterpart, although, only one sensor is used, the F-number of single aperture counterpart is the same as that of the multi-aperture system. Therefore, the number of signal electrons should be the same for the single aperture counterpart and the multi-aperture system. So the optical gain of single aperture counterpart is M (M = 9). In the selective averaging, the optical gain depends on the noise distribution, and varies from 1 to 9.

**Figure 6.**
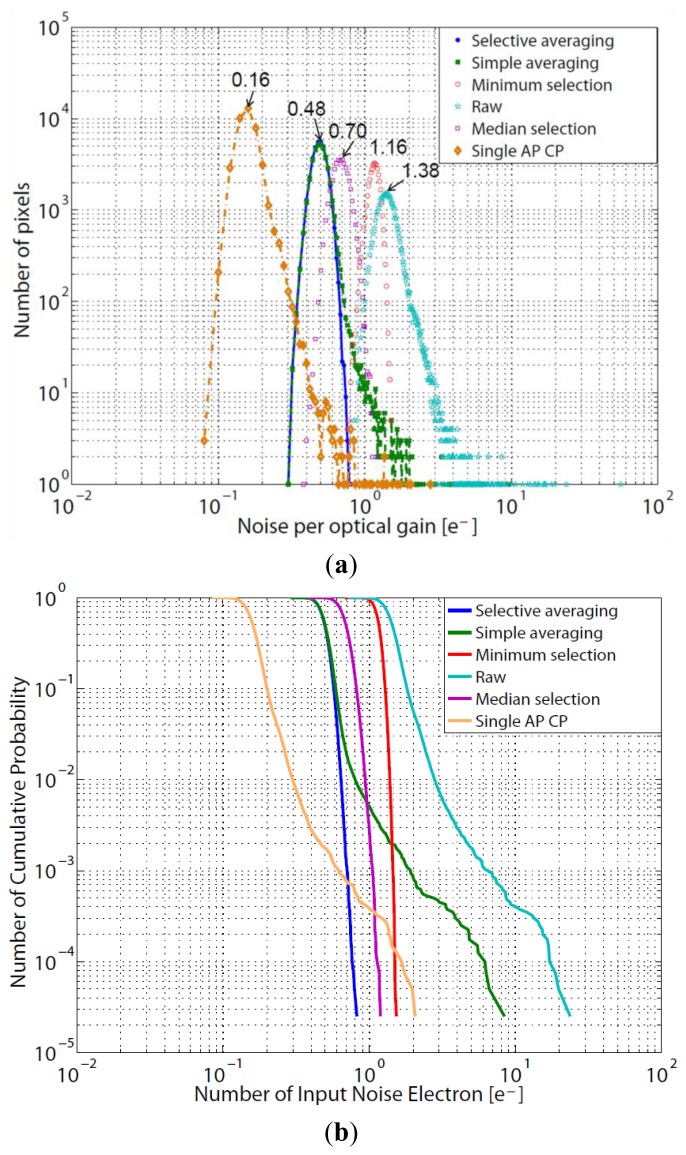
Noise distribution for different methods. (**a**) Histogram (**b**) Cumulative probability (AP: aperture, CP: counterpart).

**Figure 7.**
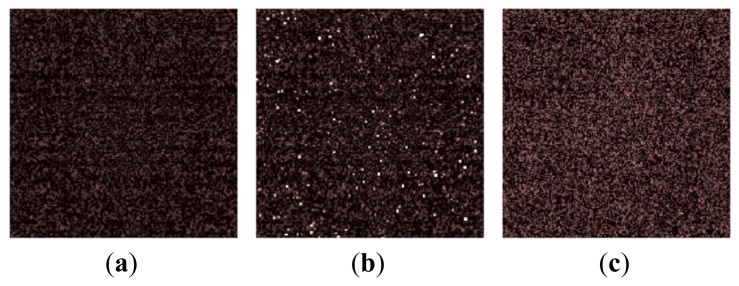

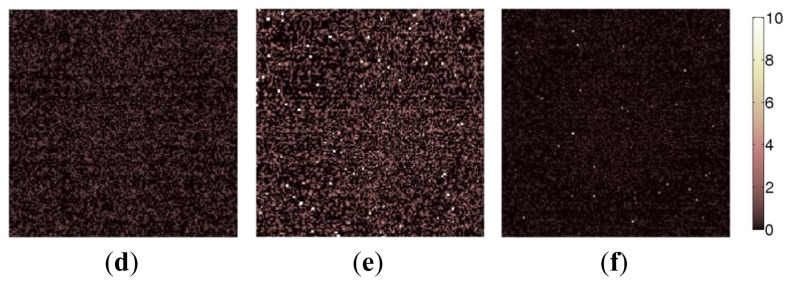
Dark image for different methods. (**a**) Selective averaging. (**b**) Simple averaging. (**c**) Minimum selection. (**d**) Median selection. (**e**) Raw. (**f**) Single aperture counterpart.

Figures 6 and 7 show the noise distribution and resultant images in the dark. The white spots represent the RTS noise or large dark current white defects. The larger the noise is, the brighter the spot is. As mentioned above, the raw data shows the original sensor noise level. The others show the noise in the reproduced images, where the sensor noise is the same. The noise of the raw shows the highest level, and tailing in the right side is caused by the RTS noise and dark current shot noise. Because of the optical gain of 9 and the sensor noise gain factor of unity which is the same as “raw”, the noise for the single aperture counterpart becomes the smallest when the noise is divided by optical gain.

As shown in Figure 6, the peak noise levels in histogram for the selective averaging and the simple averaging are the same and smaller than the minimum selection and the raw. However, in the simple averaging method, the large noise tailing such as RTS noise and dark current shot noise cannot be removed because they are included in averaging. While the large noise tailing is reduced by the median selection and minimum selection methods, the peak noise levels are larger than that of selective averaging due to unity optical gain. The selective averaging method shows the smallest peak noise among several methods except the single aperture counterpart and no large noise tailing is observed, which means that the large RTS noise and dark current shot noise are removed.

Future CISs with much finer pixel pitch will show much larger RTS noise, so that the noise tailing for the single aperture counterpart will spread more widely beyond the noise distribution of the selective averaging. Such a situation is observed in Figure 6b. Although the peak noise of the single aperture counterpart is the smallest, there are pixels with larger noise than in the selective averaging, the median selection, and the minimum selection. Therefore, it will be expected that the selective averaging method can give the best image quality in future CISs.

The numbers and percentages for each number of the selected apertures are shown in Table 1. The average number of the selected pixels is 8.35, which means the S/N of the reproduced image is significantly improved by the averaging effect. About 93% of the pixels are utilized in reproduction. As shown in Table 1, more than half of the pixels select all apertures. The number of selected pixels correlates to the amount of sensor noise. If the noise histogram has a longer tail in a finer technology, the number of selected apertures will become smaller.

## 5. Experiments

### 5.1. Image Reproduction Procedure

In the selective averaging method, the pixel value of the reproduced image is averaged using the pixel values of the selected apertures for each pixel. There are slight differences in the focal length, the skew of lens, and the lens positions among the apertures, which should be extracted with camera calibration and compensated. To identify the aperture images, they are standardized. In the standardization operation, the distortion is removed at first. Then, the focal lengths for each aperture are equalized, and the images are cropped to have the same size.

#### 5.1.1. Standardization

The standardization is composed of the following steps.

- Undistortion
- Resizing
- Cropping

##### A. Undistortion

Lens skew and distortion cause major geometric disorder on image. In the image reproduction, because the selected apertures are averaged, the images of the apertures have to be completely consistent. As the first step for this purpose, images are undistorted with the extracted distortion parameters.

##### B. Resizing

**Figure 8.**
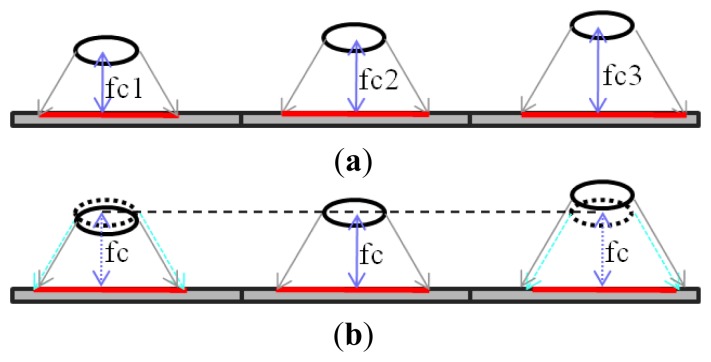
Image resize. (**a**) Different focal lengths. (**b**) After resizing.

In the multi-aperture camera, the focal length in each aperture has a slight difference to the other. There are many causes of the difference, such as manufacturing, assembling, and so on. As shown in Figure 8a, the longer the focal length is, the larger the image becomes. In the multi-aperture system, one aperture is chosen as the reference, and the images of other apertures are resized to fit the reference image. The resize scale is defined as the ratio of focal length (*fc_i_*) to the reference focal length (*fc*):

(8) $$\text{Scal}e_{i}=\frac{fc_{i}}{fc}$$

##### C. Cropping

Cropping is performed to compensate the differences of principal point and disparities among the apertures. The principle point is the position of image center, and disparity is measured based on it. Image with the same size are cropped for every aperture, where the principal points or those plus disparities should be identical. The cropped images from the multi-aperture image become identical after standardization. Those images are used to reproduce the final image by selective averaging.

#### 5.1.2. Image Procedure

**Figure 9.**
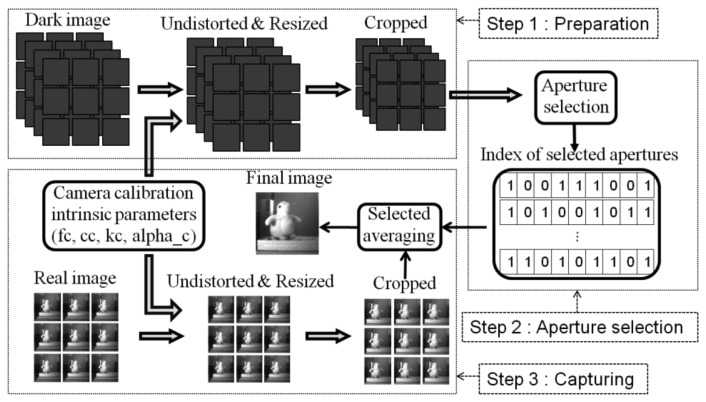
Flow chart of single image reproduction with selective averaging in multi-aperture camera.

Figure 9 shows the processing flow to reproduce a single image in the multi-aperture camera. In the preparation step, *n*-frame dark images are captured. Then, they are standardized with the above processing.

In the aperture selection step, the standardized images are used to determine the selected apertures for every pixel by the selective averaging method. Indices “1” and “0” mean that the aperture is selected and is not selected, respectively. One row of the index table corresponds to one pixel in the reproduce image, and it has nine binary digits for the nine apertures.

In the capturing step, the same standardization is operated to get the same-sized registered images for every aperture. Using the index table, which is calculated in the preparation step, selective averaging is applied pixel by pixel to produce the final image.

The preparation step is operated once after constructing the camera or image capturing because the variance of pixel noise in a dark condition and the camera parameters do not change in a short term as long as working environment is the same. However, the aperture selection step should be operated when the distance of subject changes. Because disparity is dependent on a subject distance, which means the index table has to be recalculated when the distance changes. Although, the sensor noise does not significantly change at around the room temperature, dark currents are very susceptive to temperature. Therefore, the noise variance changes due to the change in dark current shot noise. This issue can be solved with preparing several noise images for different temperatures or with thermal stabilization of the image sensors.

In the standardization, interpolation, that is averaging of pixel values among the adjacent pixels, is used. Therefore, the noise is reduced apparently, instead the spatial resolution is degraded a little. Table 2 shows the peak of noise histogram in each method with and without standardization. The noise becomes smaller after standardization. In standardization, erroneous pixel values will spread to their adjacent pixels. However, such pixel values will be automatically removed by the selective averaging, which is because the image of the deviation of noise is also interpolated in the same way as normal images in aperture selection. The amount of the effective noise of pixel in the reproduced image is considered even when interpolation is applied.

The data bandwidth and required memory size of frame buffers including the camera image, variance, standardized images, index of selection, and final image buffer in each method are shown in Table 3. This table shows the maximum date size for monochrome images. Further memory saving will be possible with resource sharing techniques. Here, H × V is the pixel count, M is the number of aperture, b is bit per pixel, and f is frame per second.

### 5.2. Results

**Figure 10.**
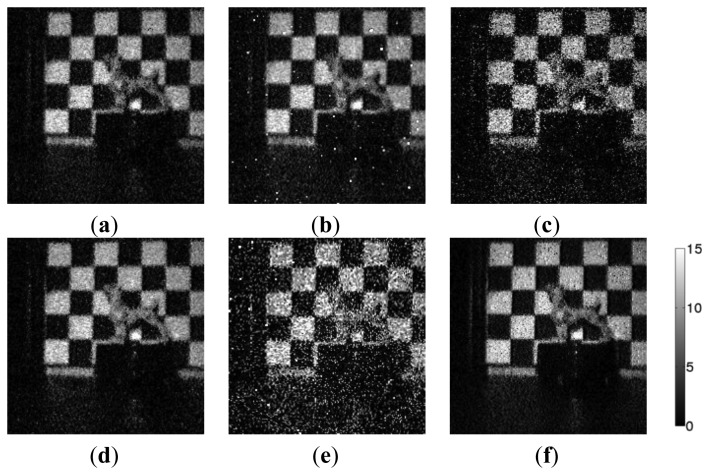
Reproduced images and reference image. (**a**) Selective averaging. (**b**) Simple averaging. (**c**) Minimum selection. (**d**) Median selection. (**e**) Raw. (**f**) Reference.

In the experiments, image registration is done manually. The cropped area is adjusted to obtain a sharp reproduced image. This procedure can be automated based on an image matching technique. Every algorithm is implemented on MATLAB. In the experiments, achromatic lenses with a focal length of 3 mm and F-number of 3.0 are utilized. Figure 10 shows the reproduced images for several methods and a synthetic reference image when the camera works under a low-light condition. The image of selective averaging shows the high quality compared with those of other methods. The reference image is synthesized by averaging 1,000 frames of one aperture image (the raw image) which are captured at 10 °C, in that condition the dark current is significantly reduced. Due to the photon shot noise is very large in a bright environment, the dominant noise in the CIS is the photon shot noise. However, in a low-light condition, RTS noise or dark current becomes dominant. The illumination on the subject is 0.04 lx, and the maximum average signal is around 11e^-^.

Table 4 shows the peak signal-to-noise ratio (PSNR) of the images in different methods and raw image when compared with the reference image. The selective averaging shows the largest PSNR value, and its improvement based on the raw image is 6.3 dB. Because the simple averaging method cannot remove the large RTS noise and dark current, the PSNR of the simple averaging image is smaller than that of the selective averaging.

**Figure 11.**
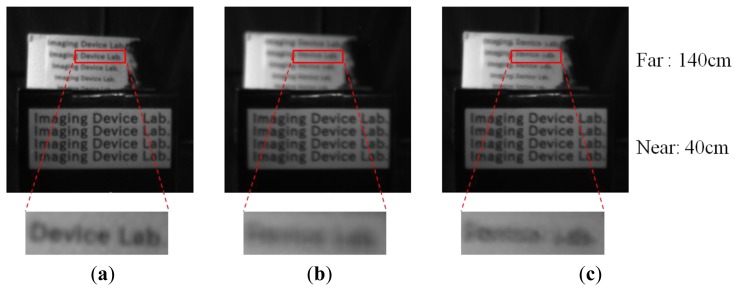
Comparison of. (**a**) Raw and virtually defocused image (**b**) Selective averaging and (**c**) Median selection.

Although the results for selective averaging and median selection give almost the same PSNR, selective averaging is preferable in terms of natural defocus emulation. As the multi-aperture system has disparity on the multi-aperture image, reproduced images become blurry when the real and a reproduction distance are different, which is called virtual defocus here. The defocus images of selective averaging, median selection and raw are shown in Figure 11, where, the object distance of the near letters is 40 cm, and that of the far letters is 140 cm. If the median selection method is applied in multi-aperture system, artifacts are generated in the virtually defocused regions, because median selection doesn't have an averaging effect. While in the selective averaging method, the reproduced image becomes naturally blurred in the virtually defocused regions due to its averaging effect.

## 6. Conclusions

In this paper, the RTS noise and dark current reduction based on a multi-aperture imaging system with a selective averaging method is presented. The architecture of the multi-aperture imaging system and the principle of the selective averaging method are discussed. In the simulation, the effective noise per optical gain is reduced from 1.38e^-^ to 0.48e^-^ in the peak of the histogram. The average number of the selected apertures is 8.35. In the experiment, the PSNR of the selective averaging image is increased of 6.3 dB. Selective averaging shows the best quality.

## Figures and Tables

**Table 1.** Number of pixels in each number of selected apertures.

**Number of Selected Apertures**	**Number of Pixels**	**Percentage [%]**
**1∼4**	0	0
**5**	30	0.1
**6**	688	1.7
**7**	4,663	11.7
**8**	14,405	36.0
**9**	20,214	50.5

**Table 2.** The peak of noise histogram in each method (dark condition).

**Method**	**Selective Averaging**	**Simple Averaging**	**Minimum Selection**	**Median Selection**	**Raw**	**Single Aperture Counterpart**
**Peak noise w standardization [e^-^]**	0.30	0.30	0.68	0.40	0.81	0.09
**Peak noise w/o standardization [e^-^]**	0.48	0.48	1.16	0.70	1.38	0.16

**Table 3.** The data bandwidth and required memory size of frame buffer.

	**Selective Averaging**	**Simple Averaging & Median Selection**	**Minimum Selection**	**Raw & Single Aperture Counterpart**
Data bandwidth [bit/s]	H × V × M × b_i_ × f	H × V × M × b_i_ × f	H × V× M × b_i_× f	H × V× b_i_ × f
Camera image [bit]	H × V × M × b_i_	H × V × M × b_i_	H × V × M × b_i_	H × V × b_i_
Variance [bit]	H × V × M × b_v_	---	H × V × M × b_v_	---
Standardized image [bit]	H × V × M × b_i_	H × V × M × b_i_	H × V × M × b_i_	H × V × b_i_
Index of selection [bit]	H × V × M	---	H × V × 1	---
Final image [bit]	H × V × b_i_	H × V × b_i_	H × V × b_i_	H × V × b_i_

**Table 4.** PSNR of the image in each method and raw.

**Method**	**Selective Averaging**	**Simple Averaging**	**Minimum Selection**	**Median Selection**	**Raw**
**PSNR [dB]**	18.70	18.02	14.59	18.57	12.41
